# The effectiveness of one “physical education minute” during lessons to develop concentration in 8- to 10-year-old schoolchildren

**DOI:** 10.3389/fspor.2023.1283296

**Published:** 2023-11-10

**Authors:** Georgiy Polevoy, Florin Cazan, Johnny Padulo, Luca Paolo Ardigò

**Affiliations:** ^1^ Department of Physical Education, Moscow Polytechnic University, Moscow, Russia; ^2^ Federal State Budgetary Educational Institution of Higher Education, Vyatka State University, Kirov, Russia; ^3^ Faculty of Physical Education and Sport, Ovidius University of Constanta, Constantia, Romania; ^4^ Department of Biomedical Sciences for Health (SCIBIS), Università Degli Studi di Milano, Milan, Italy; ^5^ Department of Teacher Education, NLA University College, Oslo, Norway

**Keywords:** schoolchildren, lesson, physical education, health, concentration

## Abstract

The demands of the school day, which includes multiple lessons, require sustained attention from students; this can be challenging, especially for young children. Concentration of attention is a critical cognitive function that impacts learning. This study involved 129 healthy schoolchildren aged 8–10 years (spanning grades 2 and 3) from a regular secondary school in Kirov, Russian Federation. A “physical education minute” (PEM), consisting of various physical exercises, was introduced during the middle of a regular lesson for the experimental group (EG), while the control group (CG) continued with their regular studies. Concentration and attention were assessed before and after the PEM using the Bourdon test. The Bourdon test results indicated a significant decrease in concentration during the lesson for the CG (*p* < 0.001), while the EG exhibited significant improvement in concentration after the PEM (*p* < 0.001). The effect size was large, demonstrating a substantial impact of this break for physical activity on concentration. It can be concluded that implementing a set of exercises in the form of a PEM in the middle of a lesson significantly improves concentration among students aged 8–10. This study underscores the effectiveness of integrating short breaks for physical activity into the daily classroom routine, ultimately benefiting students' attention, learning, and overall educational experience. Further research could explore additional factors affecting concentration and the long-term effects of the PEM on cognitive development.

## 1. Introduction

Human health constantly plays a prominent role in numerous studies. The problem of schoolchildren's health and physical development is particularly pertinent (1–3). Most authors underline the importance of a culture of physical activity in schools for the health and well-being of students (4, 5). Children in grades 2 and 3 have 5–6 lessons every day, according to the curriculum. Math, their native language, a foreign language, the world around them, and other subjects are covered; each class is 40 min long. Children must listen to the teacher, memorize the instructional material, and complete various tasks throughout the course of each class. All of this necessitates a high level of concentration. Naturally, children grow fatigued in the classroom, particularly elementary school students. At this age, it is difficult for them to maintain optimum concentration throughout the class. Concentration of attention is a feature of attention that refers to the retention of information about an item in short-term memory. Concentration is essential for task completion, especially when dealing with a novel activity. A person with high attention levels completes work more quickly and more creatively, enhancing their overall productivity. They also operate more accurately and with fewer errors. The importance of concentration of attention at school (specifically, the ability of pupils to concentrate their attention) lies in the fact that it allows pupils to better digest instructional material (6, 7). Physical education sessions are crucial, not only for the development of physical skills, but also for the so-called “discharge” of pupils' energy, which is needed when they are distracted from their regular lessons and become “charged up” with physical and psychological energy (4). However, the academic curriculum does not provide for daily instruction in physical education (8).

Physical activity is recognized as an important component of overall human culture, and this aspect of culture encompasses the components of physical education, sports, physical recreation, physical rehabilitation, and physical stabilization (9). The “physical education minute” or “physical culture pause” is one available component of physical activity culture. This brief activity usually lasts one and a half to two minutes and is held in the middle of the class period. Its primary objective is to divert pupils' attention away from the educational process and to relax their muscles, joints, spine, and eyes.

A “physical education minute” is a brief period of physical exercise implemented to prevent weariness and restore mental function (by stimulating parts of the cerebral cortex that were not participating in prior activities, while resting those that were). There have been several studies conducted in this area. They discuss the significance of this type of physical education minute for school-aged children (10, 11). However, there has been little research to prove the value of a physical education minute in the development of attentional focus in schoolchildren (12). Thus, the goal of our research was to examine the impact of a physical education minute on markers of schoolchildren's concentration levels.

## 2. Materials and methods

### 2.1. Participants

This research involved children aged 8–10 years who were studying at ordinary secondary school number 60 in the city of Kirov, the Russian Federation. An *a priori* power analysis was conducted using the G*Power software package (Version 3.1.9.4, University of Kiel, Kiel, Germany) to determine the required sample size, with α set to 0.05 and power (1−*β*) set to 0.80. The analysis revealed that a total sample size of 86 subjects would be sufficient to identify significant differences with an actual power of 0.82. A total of 129 schoolchildren took part in the study (14 [13%] of whom were left-handed, based on a handedness questionnaire (13)). The participants were boys and girls from grades 2 (65 students) and 3 (64 students). The inclusion criteria specified that all the children must be healthy and able to exercise. However, children who had health limitations and did not take part in regular physical education lessons also took part in this research, since participation in a physical education minute does not require special physical training or a high level of development of physical abilities. Half of the children from grades 2 and 3 were assigned to the control group (CG) and the remaining children were assigned to the experimental group (EG). All procedures met the ethical standards of the 1964 Declaration of Helsinki and were approved by the Research Ethics Committee of Vyatka State University (January 17, 2022 №1). Informed consent was obtained from all the parents of the adolescents included in the study.

### 2.2. Research procedure

The study was conducted at a regular school in October 2022. The duration of lessons in grades 2 and 3, according to the general education program at the school, was 40 min.

We consider the most logical timing for the minute of exercise to be the middle of the lesson (12). The children have sufficient strength and energy for the first part of the lesson; conversely, it makes no sense to spend a minute on physical activity at the end of the lesson, because the children will change to a new activity (namely, some active rest) in a few minutes' time. Thus, the physical education minute was implemented in the middle of a regular lesson and on a day when the children did not have a physical education lesson according to the school timetable.

To engage in a physical education minute, schoolchildren left their classroom for an available adjacent area and performed physical exercises (stretching, bending, up-and-down arm movements, squatting, jumping, walking on the spot, and other exercises). An example of a physical education minute is one consisting of an activity named “Three bears,” with the following supporting song:

Three bears were walking home (children waddle on the spot),

Dad was big and big (they raise their hands above their head and pull up),

Mom was smaller than him (arms at chest level),

And the little son even smaller (sit down),

He was very small (sitting down and swinging from side to side),

I walked with rattles (children stand up with hands clenched into fists in front of their chest),

And ding-ding, ding-ding (children imitate a game with rattles).

There are a number of examples of physical education minutes. At the same time, any set of exercises can be supplemented, expanded, and modified. The main objective is to distract students from the educational process, to “awaken” them, to activate them, and to attract their attention.

In the present study, 20 min after the beginning of the lesson, all the children completed a control Bourdon test together in their classroom for 1 min; this test assesses their level of development in terms of concentration and attention. Next, the children in the e.g., performed a “physical education minute” set of exercises for 1.5–2 min in an adjacent area, whereas the children from the CG continued to study the educational material. Immediately after this period of time, all the students together completed the Bourdon test again in their classroom for 1 min. Thus, all children completed the Bourdon test twice during a single lesson, such that this test showed the effect of a physical education minute on concentration and attention among children in the e.g., whereas in children from the CG it could indicate so-called adaptation or habituation to this test.

The (Benjamin) Bourdon test (with numbers) is a well-known test that has been repeatedly and positively evaluated over time in terms of its validity and reliability (14, 15). In this test, a set of numbers from 0 to 9 is presented. Its features are (14, 15):

1. There must be exactly 15 numbers per row (the number of rows is unlimited),
2. The numbers are arranged in any order, and
3. There should be 3 numbers in each row that the test-taker could cross out.

A partial example of a Bourdon test grid is presented in Table 1.

**Table 1:** Bourdon test.

4 6 3 6 5 4 8 5 2 3 6 5 4 7 8 9 6 5 2 7 8 9 6 5 4 1 2 0 3 4 1 5 2 6 9 8 5 2 0 4 5 6 3 0 1 4 5 8 7 4 0 2 5 3 6 4 0 5 9 7 0 2 4 6 3 0 2 4 5 5 2 3 6 5 4 7 8 9 6 5 2 5 8 9 6 5 4 1 2 0 5 4 1 0 2 6 9 8 5 2 0 4 5 6 3 0 1 4 5 8 7 4 0 2 5 3 6 4 0 5 9 7 0 2 4 6 5 0 2 5 2 3 6 5 4 7 8 9 6 5 2 5 8 9 6 5 4 1 2 0 5 4 1 2 0 5 4 3 7 8

The essence of the test is: within 1 min and at maximum speed, students must cross out any number that the teacher calls, for example, the number 5. At the end of 1 min, the student receives a score.

The results are processed according to the formula *C* = *R*/*E*, where

*C* = The concentration and attention score,

*R* = The number of rows attempted, and

*E* = The number of errors, including omissions and incorrectly crossed out numbers.

The results are interpreted as follows: the higher the final figure, the higher the student's concentration and attention score. This test has no set numerical values. If the student has switched to a new line, but the time is up, this line is not fully completed and is not counted in the final result.

To ensure the reliability of the results of the indicators for children in these grades, the protocol was administered twice during October 2022. For this study, days when there was no physical education for the grades included were selected, namely Monday, Wednesday, and Friday. On Tuesday and Thursday, physical education was timetabled for grades 2 and 3.

### 2.3. Statistical analysis

The results are presented in the form of means and standard deviations. Statistical analyses were conducted using SPSS 20.0 (IBM, Armonk, USA). The normality of the data was assessed by means of the Kolmogorov–Smirnov test (with a threshold of *p* < 0.05). The Kolmogorov–Smirnov test indicated a non-normal distribution for Bourdon test scores (*p* < 0.0001, *D* = 0.24033 for both groups). The reliability of the measurements (16) was assessed in the form of the intra-class correlation coefficient (ICC). The Student's t-test was used to test for significance differences between groups at baseline. Between-group differences in changes in Bourdon test score over time were analyzed using the Mann–Whitney test; differences between post-test and baseline scores for each group were assessed using the Wilcoxon test. The magnitude of differences was interpreted using the standardized effect size [Cohen's d (17)]: < 0.1 was taken to indicate no effect, 0.20–0.40 a small effect, 0.50–0.70 an effect of intermediate size, and 0.80–1.0 a large effect. The statistical significance threshold was set at *p* < 0.05.

## 3. Results

ICC was 0.887 (95% confidence interval: 0.849–0.917, *p* < 0.001). The Mann–Whitney *U* statistic was 128.500, with z-score −9.299, *p* < 0.001, and Cohen's d = 2.756 (large effect). At baseline, a t-test showed no significance difference (*p* = 0.071) between the two groups. A Wilcoxon test showed a significant change for both groups (*p* < 0.001) in the post-intervention Bourdon test compared with the baseline score. Furthermore, there was a difference between the two group in the changes that occurred. In the CG, there was a decrease in Bourdon test score (−4.0%), whereas in the e.g., test scores increased (+13.5%) when comparing post-test (9.86 ± 0.64 a.u. and 11.17 ± 0.75 a.u. for the CG and e.g., respectively) and baseline scores (10.88 ± 1.07 a.u. and 10.42 ± 1.00 a.u. for the CG and e.g., respectively).

## 4. Discussion

The effectiveness of “physical education minutes” during ordinary lessons at school has been proven by some experts. Researchers have noted that a physical education minute improves blood circulation, relieves fatigue in the muscles and nervous system, activates children's thinking, creates positive emotions, and increases their interest in classes. The optimal timing for a physical education minute is in the middle of a lesson, when children's attention is decreasing and fatigue setting in (10, 11).

Here, the influence of a physical education minute on concentration of attention in 8- to 10-year-olds was investigated for the first time. According to the results of this research, this influence is positive in second- and third-graders considered together.

Data from in children in the CG indicate that children's concentration decreases during a lesson. This is proven by the results in the CG in all three trials conducted from the beginning to the end of the experiment. The overall group scores decreased on Monday, Wednesday, and Friday. This may also indicate that children did not show adaptation (habituation) to the test between their first and second attempts to complete it, since their scores would have increased in this case. In our case, in the CG, the scores decreased between attempts in all trials.

In the e.g., scores improved between the pre-test (before the start of the minute of exercise) and the post-test (after its completion) in all trials. This indicates that the introduction of a physical education minute in the educational process is an effective means of improving concentration of attention in schoolchildren aged 8–10 years.

It should be noted that a number of studies have reported on the beneficial influence of physical activity on indicators of attention, thinking, and cognitive abilities. However, as a rule, these studies have involved either fully-fledged physical education lessons at school or sports research (18, 19). In our case, the influence of a physical education minute on concentration of attention in 8- to 10-year-olds was examined. According to the results obtained by applying the Benjamin Bourdon test, this effect turned out to be positive.

Within the existing literature, there are no publications replicating our research exactly. However, Geji et al. (20) showed that short bouts of aerobic exercise improved subsequent cognitive performance in adolescents by influencing their inhibitory control. Furthermore, Siersbaek et al. (21) found that participation in sport during leisure time improved working memory, the ability to begin an activity, and the ability to generate ideas, responses, or problem-solving strategies in first-graders.

A limitation of this study was the lack of assessment of the development quotient of participants (22). Furthermore, only a correlation was identified between PEM administration and the concentration test, prompting further research on additional co-determinants of concentration.

Of course, the design of this study could be expanded and supplemented by studying the effect of a physical education minute on memory, thinking, and other mental processes by applying a number of other mental tests. However, this study and its results scientifically prove the effectiveness of the use of a physical education minute in the educational process for schoolchildren aged 8–10 years.

## 5. Conclusions

When students aged 8–10 years participated in a set of exercises forming a “physical education minute” in the middle of a lesson, their concentration indicators improved significantly. The present study has demonstrated the effectiveness of the physical education minute in regular lessons at school. It is recommended that a small set of exercises be performed for one and a half or two minutes in the middle of the lesson. Following this, children's concentration of attention will improve, which is important for further learning and processing of educational material.

## Data Availability

The raw data supporting the conclusions of this article will be made available by the authors, without undue reservation.
